# The Uncertainty Propagation for Carbon Atomic Interactions in Graphene under Resonant Vibration Based on Stochastic Finite Element Model

**DOI:** 10.3390/ma15103679

**Published:** 2022-05-20

**Authors:** Jiajia Shi, Liu Chu, Chao Ma, Robin Braun

**Affiliations:** 1 School of Transportation and Civil Engineering, Nantong University, Nantong 226019, China; shijj@ntu.edu.cn; 2 School of Information Science and Technology, Nantong University, Nantong 226019, China; mc20171006@163.com; 3 Faculty of Engineering and Information Technology, University of Technology, Ultimo, Sydney, NSW 2007, Australia; robin.braun@uts.edu.au

**Keywords:** uncertainty quantification, graphene, carbon atomic interactions, stochastic finite element model

## Abstract

Graphene is one of the most promising two-dimensional nanomaterials with broad applications in many fields. However, the variations and fluctuations in the material and geometrical properties are challenging issues that require more concern. In order to quantify uncertainty and analyze the impacts of uncertainty, a stochastic finite element model (SFEM) is proposed to propagate uncertainty for carbon atomic interactions under resonant vibration. Compared with the conventional truss or beam finite element models, both carbon atoms and carbon covalent bonds are considered by introducing plane elements. In addition, the determined values of the material and geometrical parameters are expanded into the related interval ranges with uniform probability density distributions. Based on the SFEM, the uncertainty propagation is performed by the Monte Carlo stochastic sampling process, and the resonant frequencies of graphene are provided by finite element computation. Furthermore, the correlation coefficients of characteristic parameters are computed based on the database of SFEM. The vibration modes of graphene with the extreme geometrical values are also provided and analyzed. According to the computed results, the minimum and maximum values of the first resonant frequency are 0.2131 and 16.894 THz, respectively, and the variance is 2.5899 THz. The proposed SFEM is an effective method to propagate uncertainty and analyze the impacts of uncertainty in the carbon atomic interactions of graphene. The work in this paper provides an important supplement to the atomic interaction modeling in nanomaterials.

## 1. Introduction

Graphene is a two-dimensional nanomaterial with promising potential in a wide range of applications. The quantitative analysis methods for the extraordinary properties of graphene mainly include both the experimental and numerical aspects [1]. Compared with the experimental measurements, the numerical and theoretical methods are available and efficient supplements with merits in computational costs. At present, the main theoretical models and numerical methods for graphene research can be summarized into three categories: density functional theory [2,3] based on quantum mechanics, molecular dynamics [4] based on Newtonian mechanics, and finite element methods based on continuum mechanics. Density functional theory is the most commonly used method in condensed matter physics, computational chemistry, organic, inorganic, organometallic, and polymeric chemical systems [5,6,7]. However, when constructing an intrinsic model of the mechanical properties of graphene, the number of carbon atoms is still obviously limited by the performance of the computer. In addition, molecular dynamics simulations describe the state of motion of molecular systems well [8,9], but do not involve the evolution of electronic structures.

The finite element method has long-term development in terms of nonlinear problem solutions, parallel computing, dynamic motion modeling, etc. [10]. The high computational performance and fast convergence speed of the finite element method make it a competitive alternative to the numerical simulation of graphene. However, the variations and fluctuations of the material and geometrical properties are difficult to quantify and analyze, especially the uncertainty of the carbon atomic interactions [11]. Moreover, simplifying the graphene lattice to the periodic hexagon beams or truss elements neglects the identification of carbon atoms and carbon covalent bonds. It is necessary to introduce two different geometrical configuration components. In order to propagate the uncertainty of carbon atomic interactions in graphene, an advanced finite element model containing carbon atoms and covalent bonds is created and combined with the Monte Carlo stochastic sampling process.

In this paper, the stochastic finite element model (SFEM) is proposed for resonant frequency computation and uncertainty propagation. Both the carbon atoms and the carbon covalent bonds are taken into consideration. The method descriptions, including geometrical configuration, material parameters, and computational method, are presented in Section 2. Based on the database of the SFEM, the statistical results of the resonant frequency of graphene are recorded and compared with the reported literature. The correlation coefficients of geometrical and material parameters are computed by the Pearson and Spearman correlation methods. In addition, the vibration modes of graphene are also presented to observe and compare the displacements. Finally, a brief conclusion is summarized in the last section.

## 2. Method Description

### 2.1. Geometrical Configuration 

**Figure 1:**
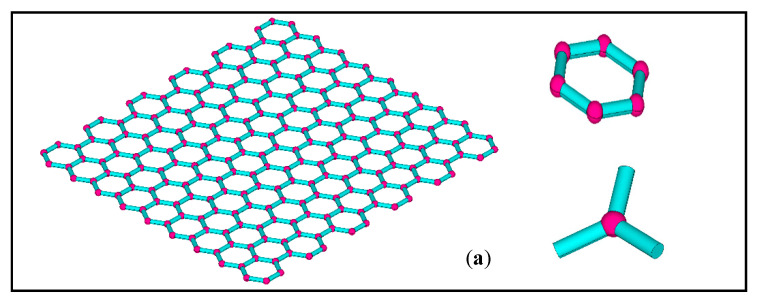

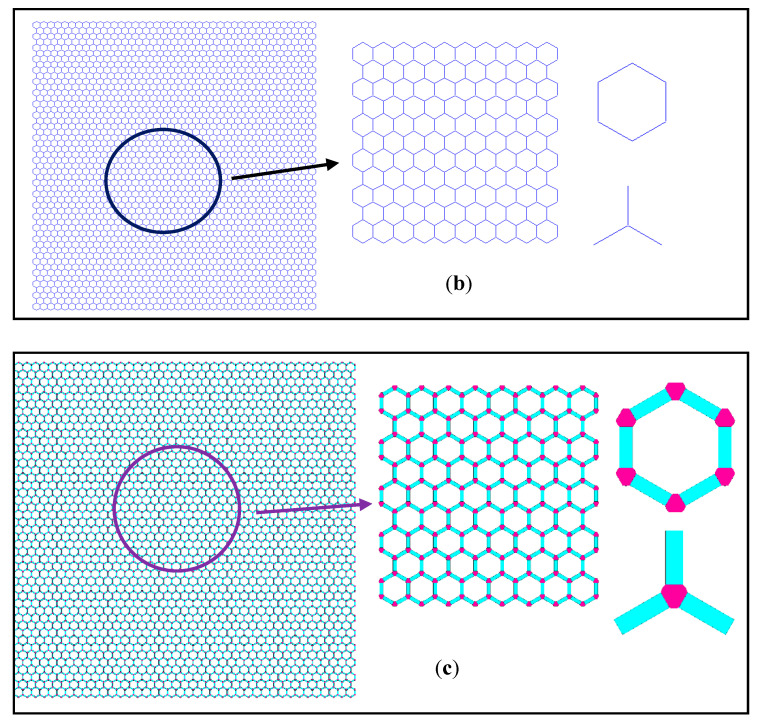
The geometrical configuration of the finite element model of graphene. (**a**–**c**) are the three-dimensional model, conventional two-dimensional model and proposed hybrid finite element model, respectively.

According to the experimental measurements and observations, the periodic honeycomb lattice of graphene is presented in Figure 1a. In general, carbon covalent bonds are simplified into trusses and beams to depict the characteristic microstructure in Figure 1b. However, the uncertainty of the carbon atomic interactions in the conventional numerical models is not taken into consideration. On the one hand, the variations and fluctuations in the geometrical and material parameters are not quantified or analyzed. On the other hand, when the atomic interaction of carbon atoms in graphene is simplified as a truss or beam element, the carbon atoms themselves disappear and are replaced by shared nodes or points. The exact role of carbon atoms and atomic interactions are neglected without a precise description. Considering the atomic interactions in graphene, this paper introduces an advanced finite plane element model, as shown in Figure 1c.

The assumptions and characteristics of the advanced finite plane element model of graphene are as follows.

**Figure 2:**
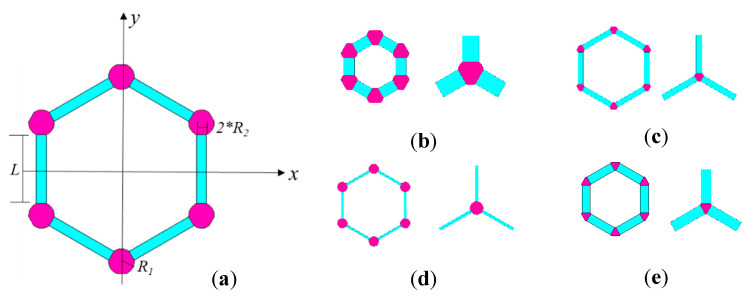
The geometrical parameters and typical examples in the finite plane element model of graphene.

(a) The finite plane element model projects the precise three-dimensional structure in Figure 1a into the two-dimensional x-y plane, which is more computationally economic than three-dimensional models, but is more sophisticated than the truss or beam finite element model;
(b) The finite plane element model is an advanced method with a similar computational competence to the truss and beam finite element model of graphene, as shown in Figure 1b. However, the finite plane element model includes not only the carbon covalent bonds but also the carbon atoms;
(c) The related geometrical parameters in the finite plane element model presented in Figure 2a are flexible to describe different special hexagons. Specifically, *L*, *R_1_*, and *R_2_* are the length of the carbon covalent bonds, the radius of the carbon atoms, and twice the width of the carbon covalent bonds, respectively;
(d) Since the carbon atoms and carbon covalent bonds in graphene are described as different geometrical components, the corresponding material parameters can be assigned to them;
(e) The carbon atoms and carbon covalent bonds, as presented in Figure 1c, share the common lines, ensuring the geometrical connection and mechanical compatibility. There will be common nodes on the shared lines after meshing the finite plane element model.

In the initial geometrical model of graphene, the values of *L*, *R_1_*, and *R_2_* are equal to 0.27 nm, 0.05 nm, and 0.032 nm, respectively. The typical examples are provided according to the changes in related geometrical parameters. For example, when *L* is as short as 0.1 nm, the period characteristic hexagon in graphene is presented in Figure 2b. When *L* is extruded to 0.4 nm, the period characteristic hexagon in graphene is presented in Figure 2c. Furthermore, the change of geometrical parameter *R_2_* also evidently impacts the configuration of the period hexagon cell in graphene and then influences the entire intrinsic lattice description. In Figure 2d,e, *R_2_* equals 0.01 nm and 0.04 nm, respectively. Therefore, the proposed geometrical configuration of the finite plane element is flexible to represent different shapes and combinations.

### 2.2. Material Parameters

In addition to the related geometrical parameters in the finite plane element model of graphene, the material parameters are listed in Table 1. According to the reported literature [10,11], the distinguished mechanical properties are measured and predicted. However, the uncertainty and obvious fluctuations of mechanical properties are the essential issues that require effective solutions. In this paper, the specific value intervals of the geometrical and material parameters are provided according to the data in the literature [10,11] and presented in Table 1.

**Figure 3:**
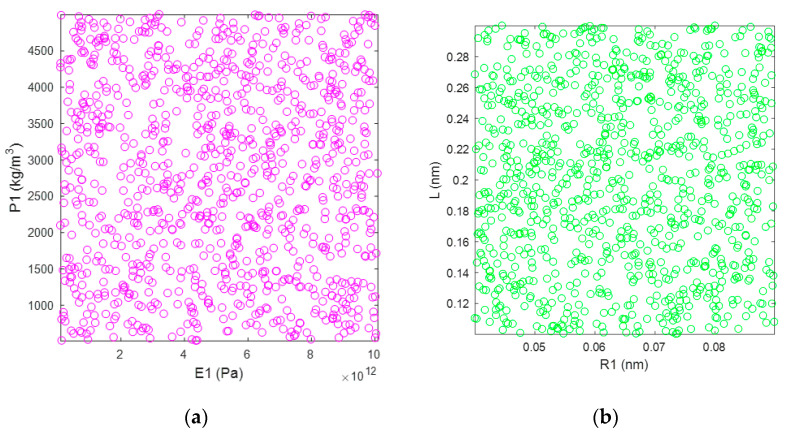
The stochastic samples of corresponding parameters in the finite plane element model of graphene based on Monte Carlo simulation. (**a**) are for the material parameters E1 and P1, (**b**) are for the geometrical parameters R1 and L, respectively.

The Monte Carlo stochastic simulation propagates the uncertainty of the parameters in the specific value intervals according to the uniform distribution. As presented in Figure 3, the stochastic samples of material and geometrical parameters in the finite plane element model are uniformly distributed in the specific interval ranges. The stochastic samples provided by the Monte Carlo simulation for material parameters of *E*_1_ and *P*_1_ are presented in Figure 3a, and those for geometrical parameters of *R*_1_ and *L* are presented in Figure 3b. In order to simplify the problem, the internal correlation between the material and geometrical parameters is supposed to be zero. The independent stochastic samples provided by the Monte Carlo simulation for the finite element computation are performed. The correlation between the corresponding parameters and the resonant frequencies of graphene is analyzed with the stochastic samples as input data and computational results of the finite element model as output data. 

### 2.3. Computational Method

**Figure 4:**
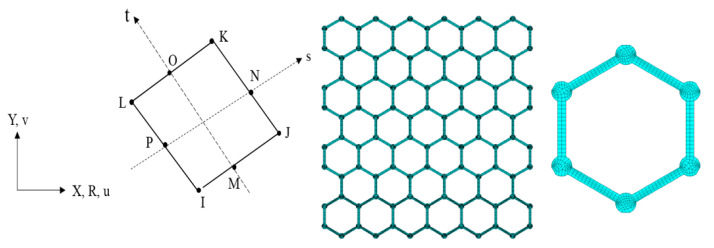
The two-dimensional 8-node quadrilateral element for the finite plane element model.

The plane element used in the finite element model of graphene is a two-dimensional 8-node quadrilateral element, as shown in Figure 4. The finite element model of graphene is meshed by the 8-node quadrilateral elements. The material parameters of carbon atoms and the covalent bonds are distributed in certain components, and the connecting lines in the different components share the common nodes. 

The shape functions of the two-dimensional 8-node quadrilateral element in the finite plane element model are expressed as,

(1) $$\begin{array}{l} u=\frac{1}{4}(u_{I}\left(1-s\right)\left(1-t\right)\left(-s-t-1\right)+u_{J}\left(1+s\right)\left(1-t\right)\left(s-t-1\right)+u_{K}\left(1+s\right)\left(1+t\right)\left(s+t-1\right)\\ \;\;\;\;\;\;\;+u_{L}\left(1-s\right)\left(1+t\right)\left(-s+t-1\right))\\ \;\;\;\;\;\;\;+\frac{1}{2}\left(u_{M}\left(1-s^{2}\right)\left(1-t\right)+u_{N}\left(1+s\right)\left(1-t^{2}\right)+u_{0}\left(1-s^{2}\right)\left(1+t\right)+u_{p}\left(1-s\right)\left(1-t^{2}\right)\right) \end{array}$$

where *u* represents the displacement. The fundamental equations are derived from the element formulations and based on the principle of virtual work [12,13],

(2) $$\int_{v}\sigma_{ij}\delta e_{ij}dV=\int_{v}f_{i}^{B}\delta u_{i}dV+\int_{s}f_{i}^{s}\delta u_{i}ds$$

where *σ_ij_* is the Cauchy stress component, $e_{ij}=\frac{1}{2}\left(\frac{\partial u_{i}}{\partial x_{j}}+\frac{\partial u_{i}}{\partial x_{j}}\right)$ is the deformation tensor, and $f_{i}^{B}$ and $f_{i}^{S}$ represent the component of body force and surface traction, respectively. *V* and *s* are the volumes of the deformed body and the surface of the deformed body on which tractions are prescribed, respectively.

The internal virtual work *W* can be indicated by:

(3) $$\delta W=\int_{v}\sigma_{ij}\delta e_{ij}dV$$

Element formulations are obtained by differentiating this virtual work expression. In derivation, only linear differential terms are kept, and all higher-order terms are ignored, so that finally a linear set of equations can be obtained. In addition, the material constitutive law is used to create the relationship between stress increment and strain increment.

(4) $${\dot{\sigma }}_{ij}^{J}={\dot{\sigma }}_{ij}-\sigma_{ik}{\dot{\omega }}_{jk}-\sigma_{jk}{\dot{\omega }}_{ik}$$

where ${\dot{\sigma }}_{ij}^{J}$ is the Jaumann rate of Cauchy stress, ${\dot{\omega }}_{ij}=\frac{1}{2}\left(\frac{\partial v_{i}}{\partial x_{j}}-\frac{\partial v_{j}}{\partial x_{i}}\right)$ is the spin tensor, and ${\dot{\sigma }}_{ij}$ is the time rate of Cauchy stress.

Therefore, the Cauchy stress rate is:

(5) $${\dot{\sigma }}_{ij}={\dot{\sigma }}_{ij}^{J}+\sigma_{ik}{\dot{\omega }}_{jk}+\sigma_{jk}{\dot{\omega }}_{ik}$$

According to the constitutive law, the stress change due to straining can be expressed as:

(6) $${\dot{\sigma }}_{ij}^{J}=c_{ijkl}d_{kl}$$

where $c_{ijkl}$ is material constitutive tensor, and the rate of deformation tensor is computed as,

(7) $$d_{ij}=\frac{1}{2}\left(\frac{\partial v_{i}}{\partial x_{j}}+\frac{\partial v_{j}}{\partial x_{i}}\right)$$

where *v_i_* is the velocity and *x_i_* is the coordinates.

The Cauchy stress rate can be written as:

(8) $${\dot{\sigma }}_{ij}=c_{ijkl}d_{kl}+\sigma_{ik}{\dot{\omega }}_{jk}+\sigma_{jk}{\dot{\omega }}_{ik}$$

The hydrostatic pressure $\bar{P}$ or volume change rate is interpolated on the element level and solved on the global level independently in the same way as displacement. The final stiffness matrix has the format of:

(9) $$\left[\begin{array}{cc} K_{uu} & K_{up}\\ K_{pu} & K_{pp} \end{array}\right]\left\{\begin{array}{c} \Delta u\\ \Delta \bar{P}\; \end{array}\right\}=\left\{\begin{array}{c} \Delta F\\ 0 \end{array}\right\}$$

where Δu is displacement increment, $\Delta \bar{P}$ is hydrostatic pressure increment.

The competitive merits of the finite element model used for the numerical investigation of graphene are the economics of computational cost and feasibility of computation of a massive number of atoms. The resonant frequency computation by the finite plane element model based on Monte Carlo stochastic simulation is programmed in the following flowchart.

**Figure 5:**
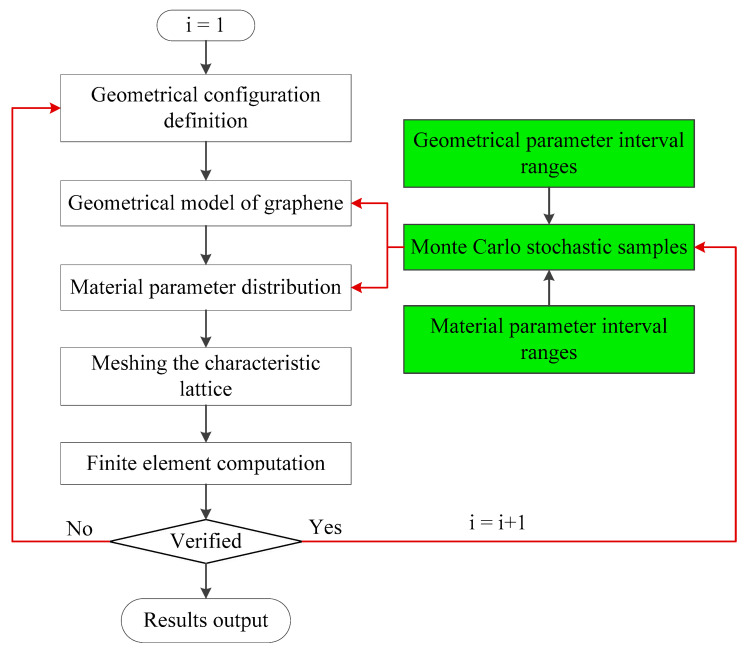
The flowchart of resonant frequency computation by the finite plane element model based on the Monte Carlo stochastic simulation.

As shown in Figure 5, the green boxes represent the Monte Carlo stochastic sampling procedure of the geometrical and material parameters. The geometrical configuration is defined above. The different values of material parameters of carbon atoms and carbon covalent bonds are distributed according to the specific interval ranges. The Monte Carlo simulation is performed to provide the unified distributed stochastic samples for the corresponding parameters. The characteristic lattice of graphene is meshed by the two-dimensional 8-node quadrilateral element. The finite element computation is performed under the ANSYS Parameter design language. The result accuracy and convergence are verified by the comparison with the reported literature [14,15,16,17,18,19,20,21,22,23]. The program loop will continue to execute until a sufficient number of Monte Carlo stochastic samples is reached.

## 3. Results and Discussion

### 3.1. Statistical Results

**Figure 6:**
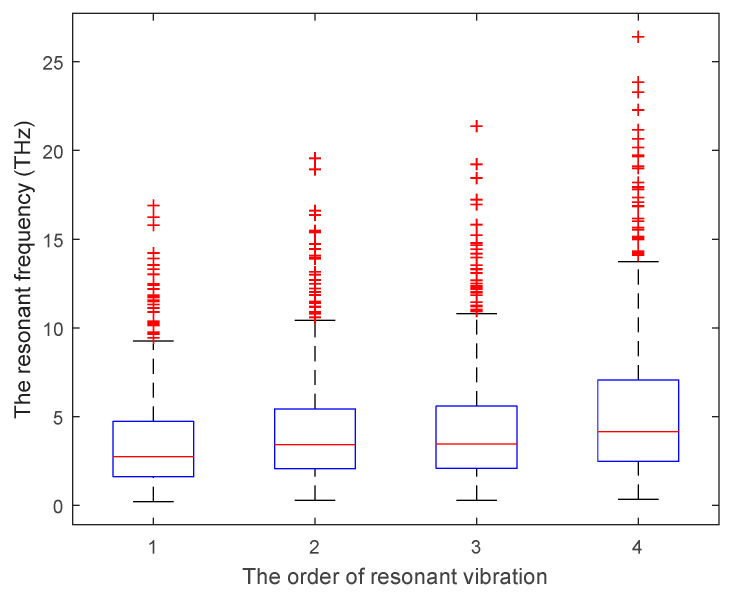
The statistic results of resonant frequency in the finite plane element model of graphene.

Based on the Monte Carlo simulation, the stochastic sampling is performed within the specific interval ranges of the geometrical and material parameters, and the statistical results are presented in Figure 6. The amplitudes of the resonant frequency of the first four vibration modes are in THz. The mean, maximum, minimum, and standard variance of the resonant frequency of graphene are recorded and compared with the results in the reported literature [14,15,16,17,18,19,20,21,22,23], as shown in Table 2.

Compared with the reported results, the mean values of the resonant frequency of the first vibration mode are evidently larger than the expected value. However, it is worth noting that the minimum resonant frequency of the first vibration mode is as small as 0.2131 THz. In addition, the maximum resonant frequency of the first vibration mode is extended to 16.894 THz. Therefore, the results in the reported references [14,15,16,17,18,19,20,21,22,23] are completely included within the result interval range of the proposed model. In addition, the statistical results of the resonant frequency of the second, third, and fourth vibration modes match the results in the reported literature well, both in the mean results and the interval ranges. Moreover, the variances of resonant frequencies are limited to 2.5–3.9 THz, even the interval ranges of resonant frequencies are amplified from the first to the fourth modes. In other words, the convergences of the proposed stochastic finite element model present satisfied property, while the risk of Monte Carlo simulation is controlled at an acceptable level. Therefore, the proposed finite plane element model of graphene is an effective alternative method for the currently available approaches in accordance with the result interval range and variances.

**Figure 7:**
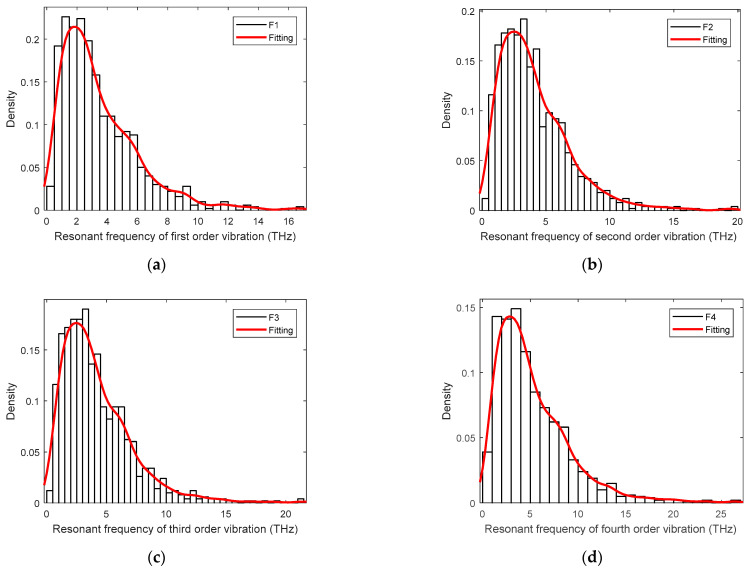
The probability density distribution of resonant frequency in the finite plane element model of graphene. (**a**–**d**) are for the first-fourth resonant vibration, respectively.

In order to describe the results more exactly, the probability density distributions of the resonant frequency of the stochastic samples are also presented in the histograms with the fitting curves, as shown in Figure 7. The uniform distribution of the input material and geometrical parameters in the finite plane element model leads to the solely concentrated peaks in the histograms of the resonant frequency. For the first vibration mode, even though the computed mean value is 3.4905 THz, the peak value of the probability density distribution in Figure 7a is located on the left side of the mean value. As a consequence, the mean value of the first vibration mode is enlarged by a few large values on the right side of the probability density distribution. Furthermore, the probability density distributions in Figure 7 all peak in the middle left.

### 3.2. Parameter Discussion

**Figure 8:**
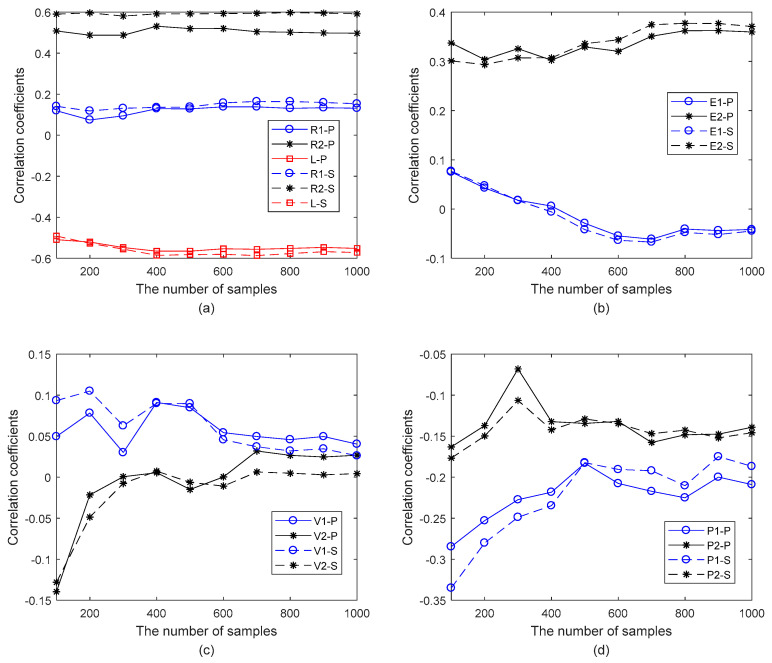
The correlation coefficients of geometrical and material parameters in the finite plane element model of graphene. (**a**) is for the geometrical parameters R1, R2 and L; (**b**) is for the material parameters E1 and E2; (**c**) is for the material parameters v1 and v2; (**d**) is for the material parameters P1 and P2.

The correlation coefficients of geometrical and material parameters in the finite plane element model of graphene are computed by the Pearson and Spearman methods, as presented in Figure 8. The correlation coefficients computed by the Pearson and Spearman methods have substantial agreements with small discrepancies. In Figure 8a, compared with the geometrical parameters, *R_1_*, *R_2_*, and *L* are the more critical factors to impact the resonant frequency of graphene. In other words, the length and the width of the carbon covalent bonds are essential in the finite plane element model of graphene. This consequence reaches good agreements with the assumption of the reported beam or truss finite element model of graphene [10,11,23]. 

In addition, the widths of the carbon covalent bonds have positive correlation coefficients, while the lengths of the carbon covalent bonds present negative values. For the negative correlation coefficients, the Young’s modulus of carbon atoms in Figure 8b, the Poisson’s ratio of the carbon covalent bonds in Figure 8c, and the mass density of carbon atoms and carbon covalent bonds in Figure 8d present negative values. It is reasonable to find the negative influences of mass density on the resonant frequency since the mass matrix is in the denominator. However, compared with the length of carbon covalent bonds, the impacts of other parameters with negative effects are smaller. 

Furthermore, the Young’s modulus of carbon covalent bonds has more evident impacts on resonant frequency than that of carbon atoms in Figure 8b. However, the mass density of carbon atoms presents more obvious influences than that of carbon atoms in Figure 8d. More importantly, with the increment of the number of stochastic samples, the correlation coefficients tend to converge to a certain value for each parameter. On the other hand, the fluctuations of Monte Carlo relative errors caused by the number of stochastic samples are also observed in Figure 8. According to the increase of the stochastic samples, the Monte Carlo relative errors in correlation computation for *R_1_*, *R_2_*, and *L* in Figure 8a are steady, and those for *E_1_* and *E_2_* in Figure 8b are steady as well. However, the Monte Carlo relative errors in correlation computation for *V_1_*, *V_2_*, *P_1_*, and *P_2_* present evident variations in Figure 8c,d. Therefore, a sufficient number of stochastic samples is necessary to ensure the correlation coefficient accuracy. 

### 3.3. Vibration Modes

In addition to the resonant frequency of graphene, the vibration modes are also computed by the finite plane element model of graphene. According to the above results, the length and width of the carbon covalent bonds are the more important factors influencing the resonant frequency of graphene. The extreme values of the geometrical parameters are introduced in the finite plane element model of graphene to compare the vibration modes. 

**Figure 9:**
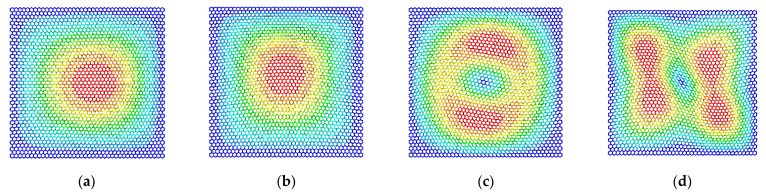
The vibration modes of graphene with the length of the carbon covalent bonds equal to 0.1 nm ((**a**–**d**) represent the first four resonant vibration modes).

**Figure 10:**
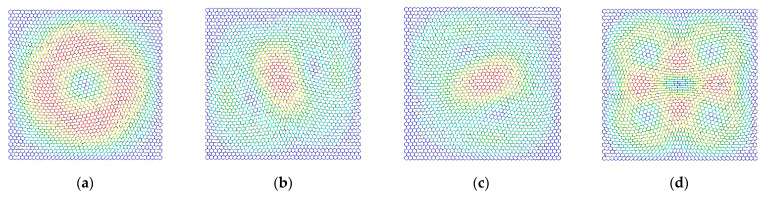
The vibration modes of graphene with the length of the carbon covalent bonds equal to 0.4 nm ((**a**–**d**) represent the first four resonant vibration modes).

**Figure 11:**
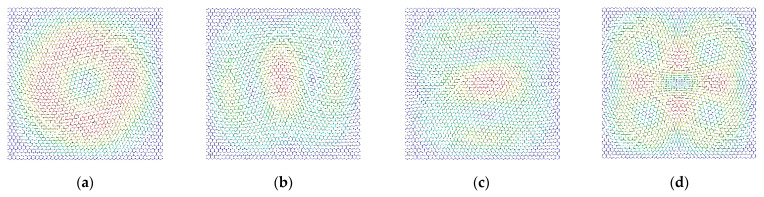
The vibration modes of graphene with the width of the carbon covalent bonds equal to 0.005 nm ((**a**–**d**) represent the first four resonant vibration modes).

**Figure 12:**
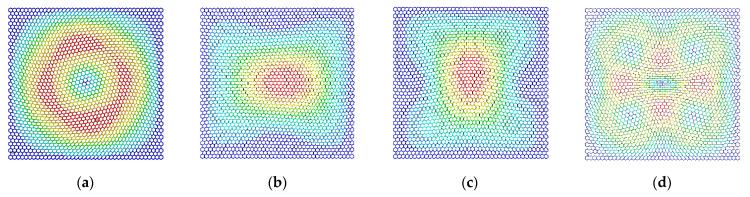
The vibration modes of graphene with the width of the carbon covalent bonds equal to 0.02 nm ((**a**–**d**) represent the first four resonant vibration modes).

The differences in the vibration modes of the first four resonant vibration modes are presented and compared, as presented in Figure 9, Figure 10, Figure 11 and Figure 12. It is evident that the displacement contour results in the first-order resonant vibration are not evident, but in the second, third, and fourth resonant vibrations, the differences are evident. Compared with the truss and beam finite element model, the proposed finite plane element model of graphene is more competitive. On the one hand, both the carbon atoms and carbon covalent bonds are considered, and the vibration modes reflect the existence of the carbon atoms in the lattice of graphene, while the conventional truss and beam finite element model simplifies the graphene into the periodic honeycomb hexagon structure. On the other hand, the two-dimensional 8-node quadrilateral element used in the proposed model provides more accurate results than that of the truss and beam finite element. With the additional middle points on each edge of the quadrilateral element, the finite plane element model is more sensitive to deformation and displacement. Especially for the common nodes shared by the carbon atoms and the carbon covalent bonds, additional middle points in each edge strengthen the mechanical compatibility. 

Furthermore, the resonant vibration modes in Figure 10 are similar to those in Figure 11. However, the discrepancies of resonant vibration modes in Figure 9 and Figure 12 are evident in others. Compared with the results in the reported literature [10,11,23], the displacement results in Figure 10 and Figure 11 are more approximated and reach good agreements in graphene vibration modes. The computational vibration modes are sensitive to the involved parameters in the proposed stochastic finite element model. The appropriate values for the corresponding parameters are the key essentials to the results’ accuracy. Therefore, the proposed finite element model not only has merits in terms of computational expense and feasibility in the massive stochastic sampling process but is also flexible in presenting the precise vibration modes of graphene, considering both carbon atoms and covalent bonds.

## 4. Conclusions

The proposed stochastic finite plane element model of graphene is an effective alternative to the currently available approaches with competitive competencies in terms of computational performances and result consistency. In short, the following key points can be concluded based on the results.

(1) The commonly shared nodes in carbon atoms and carbon covalent bonds in the two-dimensional 8-node quadrilateral element keep the geometrical connection and mechanical compatibility well.
(2) The interval ranges of resonant frequencies computed by the finite plane element model completely include the results in the reported literature.
(3) The correlation coefficients computed by the Pearson and Spearman methods have substantial agreements with small discrepancies in the geometrical and material parameters.
(4) The length and the width of the carbon covalent bonds in the finite plane element model of graphene are the essential factors that impact the resonant frequencies.
(5) The proposed finite element model not only has merits in terms of computational expense and feasibility in the massive stochastic sampling process but also is flexible in presenting the precise vibration modes of graphene with consideration of both carbon atoms and covalent bonds.

## Figures and Tables

**Table 1:** Material and geometrical parameters in the finite plane element model of graphene.

Symbols	Definitions	Value Intervals	Units
*E*_1_	The Young’s modulus of carbon atoms	10^11^–10^13^	Pa
*E*_2_	The Young’s modulus of carbon covalent bonds	10^6^–10^8^	Pa
*v*_1_	The Poisson’s ratio of carbon atoms	0.1–0.4	-
*v*_2_	The Poisson’s ratio of carbon covalent bonds	0.1–0.4	-
*P*_1_	The physical density of carbon atoms	500–5000	Kg/m^3^
*P*_2_	The physical density of carbon covalent bonds	500–5000	Kg/m^3^
*R*_1_	The radius of carbon atoms	0.04–0.09	nm
*R*_2_	The two times width of carbon covalent bonds	(0.1–0.5) ∗ *R*_1_	nm
*L*	The length of carbon covalent bonds	0.1–0.4	nm

**Table 2:** The statistic results of resonant frequency in the finite plane element model of graphene.

	*F*_1_ (THz)	*F*_2_ (THz)	*F*_3_ (THz)	*F*_4_ (THz)
Mean	3.4905	4.0902	4.1838	5.2333
Maximum	16.894	19.554	21.362	26.402
Minimum	0.2131	0.2808	0.2816	0.3319
Variance	2.5899	2.7923	2.9301	3.8381
Liu [14]	1.6081	3.7232	4.3172	6.4323
Kudin [15]	1.5818	3.6623	4.2466	6.3271
Gupta [16]	1.7581	4.0706	4.7201	7.0325
Lu [17]	1.4311	3.3135	3.8422	5.7246
Wei [18]	1.5946	3.6921	4.2811	6.3786
Cadelano [19]	1.5649	3.6232	4.2012	6.2595
Reddy [20]	1.3869	3.2111	3.7234	5.5475
Zhou [21]	1.8716	4.3334	5.0248	7.4865
Khatibi [22]	1.6030	2.4970	2.5980	3.5770
Chu [23]	1.7282	3.2925	3.7442	5.1892
